# Moderate Drought Stress Affected Root Growth and Grain Yield in Old, Modern and Newly Released Cultivars of Winter Wheat

**DOI:** 10.3389/fpls.2017.00672

**Published:** 2017-05-01

**Authors:** Yan Fang, Yanlei Du, Jun Wang, Aijiao Wu, Sheng Qiao, Bingcheng Xu, Suiqi Zhang, Kadambot H. M. Siddique, Yinglong Chen

**Affiliations:** ^1^State Key Laboratory of Soil Erosion and Dryland Farming on the Loess Plateau, Northwest A&F UniversityYangling, China; ^2^Institute of Soil and Water Conservation, Chinese Academy of Sciences and Ministry of Water ResourcesYangling, China; ^3^State Key Laboratory of Grassland Agro-ecosystems, Institute of Arid Agroecology, School of Life Sciences, Lanzhou UniversityLanzhou, China; ^4^The UWA Institute of Agriculture, and UWA School of Agriculture and Environment, The University of Western Australia, PerthWA, Australia

**Keywords:** drought stress, root mass, root length density, semi-arid Loess Plateau, *Triticum aestivum*

## Abstract

To determine root growth and grain yield of winter wheat (*Triticum aestivum* L) under moderate drought stress, a nursery experiment and a field trial were conducted with or without water stress using three representative cultivars released in different years: CW134 (old landrace), CH58 (modern cultivar) and CH1 (new release). In the nursery experiment, plants were grown in soil-filled rhizoboxes under moderate drought (MD, 55% of field capacity) or well-watered (WW, 85% of field capacity) conditions. In the field trial, plots were either rainfed (moderate drought stress) or irrigated with 30 mm of water at each of stem elongation, booting and anthesis stages (irrigated). Compared to drought stress, grain yields increased under sufficient water supply in all cultivars, particular the newly released cultivar CH1 with 70% increase in the nursery and 23% in the field. When well-watered (nursery) or irrigated (field), CH1 had the highest grain yields compared to the other two cultivars, but produced similar yield to the modern cultivar (CH58) under water-stressed (nursery) or rainfed (field) conditions. When exposed to drought stress, CW134 had the highest topsoil root dry mass in topsoil but lowest in subsoil among the cultivars at stem elongation, anthesis, and maturity, respectively; while CH1 had the lowest topsoil and highest subsoil root dry mass at respective sampling times. Topsoil root mass and root length density were negatively correlated with grain yield for the two water treatments in nursery experiment. When water was limited, subsoil root mass was positively correlated with thousand kernel weight (TKW). In the field trial, CH1 and CH58 used less water during vegetative growth than CW134, but after anthesis stage, CH1 used more water than the other two cultivars, especially in the soil profile below 100 cm, which was associated with the increased TKW. This study demonstrated that greater root mass and root length density in subsoil layers, with enhanced access to subsoil water after anthesis, contribute to high grain yield when soil water is scarce.

## Introduction

Wheat (*Triticum aestivum* L.) is the most widely grown cereal crop. The primary objective of most breeding programs is the development of high-yielding genotypes and improvement of grain yield potential (Foulkes et al., 2009). Since the Green Revolution, wheat yields have increased in many regions of the world (Gewin, 2010). To cope with the future demands for food, the yield of wheat and its yield potential under water-limited conditions needs to increase because farmland is increasingly being threatened by drought stress around the world (del Pozo et al., 2016). In addition to yield potential, yield stability (the selection of genotypes with more stable performance) remains a key objective for crop breeders (Slafer and Kernich, 1996). Becker and Leon (1988) believe that a successful genotype should produce consistently high yields over a wide range of environmental conditions, with a concomitant increase in stress tolerance (Tollenaar and Lee, 2002), such as drought stress (del Moral et al., 2003). There is evidence that modern cultivars with higher yields respond better to environmental change and have greater yield potential but less yield stability than older cultivars (Siddique et al., 1989a,b; Loss and Siddique, 1994; Fufa et al., 2005; Acreche et al., 2008). These studies focused mainly on shoot traits, with root studies often overlooked.

Drought is considered as one of the most important environmental stresses (Cattivelli et al., 2008). Improving yield under drought stress is an essential breeding target (Cattivelli et al., 2008). Drought stress can reduce wheat yields by up to 50% (Reynolds et al., 2007) due to significant reductions in plant growth and shoot production (Ehdaie et al., 2008, 2012). Several shoot-related physiological and morphological traits related to grain yield under drought conditions have been identified (Edmeades et al., 1999; Araus et al., 2002; Richards et al., 2002; Abdolshahi et al., 2015). However, root-related traits have been largely neglected by breeders due to the lack of straightforward, efficient methods for studying root systems in soil (Manschadi et al., 2006; Den Herder et al., 2010). Root system is the major plant organ for water and nutrient acquisition and influence plant growth and grain productivity (Ehdaie et al., 2012; Palta and Yang, 2014).

There are contrasting research reports on the value of the root system under drought stress for grain yield. One argument is that a relatively large root system is essential for crops grown in drought areas to absorb more soil water and relief drought stress (Palta et al., 2011; Ehdaie et al., 2012). An alternative view is that since the roots are a major sink for assimilates, reducing root mass increases the availability of assimilates for aboveground parts including grain yield (Siddique et al., 1990; Zhang et al., 1999; Song et al., 2009). Passioura (1983) and Zhu and Zhang (2013) argued that a small root system could have a positive effect on grain yield in water-limited situations. Genotypes with a large topsoil root system should be able to capture soil moisture from the topsoil during occasional spring rainfall and use it for grain filling (Palta et al., 2011; Ehdaie et al., 2012). In addition, a vigorous root system in the topsoil can absorb nutrients that are mostly concentrated in the upper layers of soil (Manske and Vlek, 2002). However, a large root mass in the topsoil layer may aggravate the effects of water stress and increase abscisic acid (ABA) levels, which would reduce stomatal conductance and photosynthesis (Ramachandra Reddy et al., 2004; Du et al., 2013). Root traits should be considered in the study of crop water-use efficiency (Raza et al., 2015).

A number of researchers reported genetic improvements in Chinese wheat (Song et al., 2009; Zhang et al., 2010; Zheng et al., 2011; Sun et al., 2014) and several studies have observed morphological and physiological changes in aboveground parts giving rise to yield increases under water-limited environments on the Loess Plateau (Zhang et al., 2009; Chen et al., 2011). A high photosynthetic rate in winter wheat was found in these areas (Sun et al., 2014). Roots play a vital role in water uptake, and thus affect photosynthetic rate, which associated with changes in yield, especially in the semi-arid areas (Reynolds et al., 2000; Sun et al., 2014). However, few studies examined winter wheat root systems in such regions. Here, three winter wheat cultivars, a newly released cultivar CH1, a modern cultivar CH58 and an old landrace CW134 (Chen et al., 2014), were selected to determine changes in the root system and the relationship between root growth and grain yield under drought and well-watered conditions. It was hypothesized that root system adaptive to drought stress was improved along with genetic improvements, and the changed root system of modern cultivar led to a higher yield stability and yield potential than the old landrace.

## Materials and Methods

### Plant Materials

Three local cultivars of winter wheat (*T. aestivum* L.) with similar plant heights were used in both rhizobox experiment in a nursery and field trial. Changwu 134 (CW134) is a widely grown old landrace and Changhan 58 (CH58) is a modern cultivar released in 2004. The both cultivars were among the most successful releases of their respective eras, based on the area cropped and cultivation period in the region. Changhang 1 (CH1) is a newly released cultivar (2014) and is the current common winter wheat cultivar grown on the Loess Plateau. The average yields of cultivars CW134, CH58 and CH1 were 4500, 5300, and 5500 kg hm^-2^ based on their cultivation by local farmers on the Loess Plateau. The three cultivars were developed for the semi-arid dryland agricultural area on the Loess Plateau, China. Seeds were obtained from the Institute of Crop Germplasm Resources, Chinese Academy of Agricultural Sciences, Beijing, China.

### Rhizobox Experiment

#### Experimental Design, Establishment, and Maintenance

A soil-filled rhizobox experiment was conducted from October 2014 to June 2015 in a nursery in Yangling (108°4′28″ E, 34°16′56″ N, 500 m a.s.l), Shaanxi Province, Northwest of China. A randomized complete block design with three wheat cultivars and two water treatments was used. Two water treatments were: (1) moderate drought stress (MD, 55% field water capacity, FWC) and well-watered (WW, 85% FWC).

Rectangular rhizoboxes (400 mm wide, 600 mm subsoil, and 30 mm thick) were constructed with stainless steel frames and clear perspex on one side (6 mm thick). Each rhizobox was filled with 12 kg of sieved (2 mm mesh) air-dried soil, collected from the upper 20 cm of a cultivated field in Yangling, a typical Calcaric Regosol (FAO/UNESCO soil classification system; Wang et al., 2010). Gravimetric moisture content at field capacity was 26% and wilting point was 9%. The soil organic matter content, total nitrogen (N) and total phosphorus (P) contents were 19.1, 0.93, and 0.88 g kg^-1^, respectively. Soil alkali-hydrolysable N was 65.0 mg kg^-1^, rapidly available phosphorus was 17.9 mg kg^-1^ and available potassium was 163.6 mg kg^-1^. Before sowing, base fertilizer, equivalent to 120 kg ha^-1^ N (0.36 g kg^-1^ N as urea) and 60 kg ha^-1^ P (0.68 g kg^-1^ of P_2_O_5_ as calcium superphosphate), was applied to the soil to ensure sufficient nutrition supply. The rhizoboxes were weighed and watered at 08:00 and 16:00 h every 2 days throughout the experimental period. Six seeds of each cultivar were planted in each rhizobox at a depth of 30 mm in October. Seedlings were thinned to three per rhizobox following emergence. After thinning, the soil in the rhizoboxes was covered with a layer of perlite to reduce water evaporation. There were six replicate rhizoboxes in each treatment and the rhizoboxes were arranged randomly in a rainout shelter.

#### Plant Sampling and Assessments

Plants were assessed at stem elongation stage (2 March) and at maturity (11 May) with three rhizoboxes for each assessment per treatment. At stem elongation stage, tiller number and leaf area (CID Inc, Camas, WA, USA) of each plant were recorded. At maturity, spike number, number of grains per rhizobox, average thousand kernel weight (TKW) and grain yield were determined. At each harvest, aboveground parts were excised at the root/shoot interface, dried for 24 h at 75°C and then weighed. The harvest index (HI) at the maturity assessment was calculated using the following equation:

HI = grain weight/total aboveground dry weight

After removal of shoot, the rhizoboxes were laid on the steel side to remove the perspex panel for sampling roots. The soil with roots in each rhizobox was separated into 0–20 cm (topsoil) and 20–60 cm (subsoil) layers. Roots in each layer were washed carefully by hand, and remove any attached soil. Washed roots were placed in plastic bags and stored at 4°C. Roots were then scanned at 600 pixels per mm and root images were analyzed using WinRHIZO (Regent Instruments Inc., Québec City, QC, Canada) as described by Himmelbauer et al. (2004). Root length density (RLD, cm cm^-3^) calculated as follows:

RLD = root length/soil volume

Scanned roots were dried in a forced-air dryer for 24 h at 75°C. Topsoil and subsoil root mass and total root mass were determined. The ratio of root to shoot mass was calculated.

### Field Experiment

#### Experimental Design, Establishment, and Maintenance

A field experiment was conducted from October 2015 to June 2016 at Changwu Agro-ecological Experiment Station of Chinese Academy of Sciences, Shaanxi Province, China (107°40′30″ E, 35°14′30″ N, 1200 m a.s.l.). The mean annual temperature is 9.1°C, and the long-term average annual precipitation is 584 mm (1957–2001), with 68% rainfall between June and September. The average frost-free period is 194 days per year. The soil is a typical Calcaric Regosol (FAO/UNESCO soil classification system; Wang et al., 2010) which consists of 59% silt, 37% clay, and 4% sand with a pH of 8.4. It has organic matter content of 11.8 g kg^-1^, total N content of 0.87 g kg^-1^, mineralized N of 3.15 mg kg^-1^, Olsen-P of 14.4 mg kg^-1^, and NH_4_OAc-extractable K of 144.6 mg kg^-1^ (Gong et al., 2007). The soil bulk density is 1.36 g cm^-3^, and the permanent wilting coefficient and field capacity is 10% and 26% (determined gravimetrically), respectively.

A randomized block design with three wheat cultivars, two water treatments was used. There were three replicate plots per treatments. Seeds were sown at 5 cm deep in rows 20 cm apart with a total density of 225 seeds m^-2^. The water treatments were (1) rainfed (RF), and (2) irrigated (IR) with 30 mm of water applied at the beginning of stem elongation (29 March), booting (28 April) and anthesis (22 May), respectively, using the flood irrigation method by measuring the amount of water applied with a flow meter.

#### Plant Sampling and Assessment

Plants were harvested at stem elongation (14 April), anthesis (15 May) and physiological maturity (25 June). In each plot, plants in 1 m^2^ were used for the assessment. At stem elongation and anthesis, 20 representative plants were sampled to measure leaf area (CID Inc, Camas, WA, USA) and tiller number. At maturity, grain yield, spike number, grain number, TKW and HI were determined. Plant tissues were dried in a forced draft oven at 75°C.

Root samples were collected at stem elongation and anthesis stages. Roots were collected from the soil profile (0–100 cm) using a root corer with a diameter of 9 cm. The soil profile was divided into two layers, 0–20 cm considered as topsoil and 20–100 cm as subsoil. Three plants per plot were selected for sampling roots. Three cores were taken for each plant with one centered over the cut stem of the sampled plants and the other two from the mid-point between the rows. The root samples were washed free of soil through a sieve with 0.4 mm mesh. The cleaned roots were placed in plastic bags and stored at 4°C, and then scanned. Root mass and RLD were measured and calculated as described for the rhizobox experiment.

#### Soil Moisture

The soil water content was measured gravimetrically. Soil moisture was recorded at sowing, stem elongation, anthesis and maturity by coring using an auger of about 9 cm diameter. The soil profile 0–200 cm was divided into 10 layers of 20 cm each and sampled in three replicates per layer. Evapotranspiration (ET, mm) and water used for a given period was estimated from:

ETc = P+ΔW

Where ΔW is the change in soil water stored in the 0–200 cm profile between two soil moisture content measurements, and P is the recorded rainfall.

## Statistical Analysis

Data of plant growth, grain yield and root traits was subjected to two-way ANOVAs to test the main effects (cultivar and water treatment) and their interactions using Statistical Analysis System (SAS 9.3). Means were separated using Fisher’s protected l.s.d. at the 5% level of significance. Associations between parameters were examined by correlation analysis.

## Results

### Rainfall and Temperature Variation in the Field Trial

**FIGURE 1:**
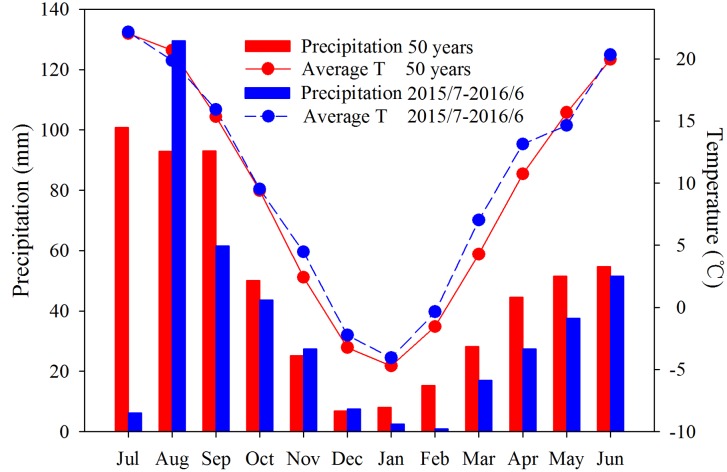
**Precipitation (bars) and temperature (lines) in 2015–2016 (blue bars and lines) and the long-term (50 years) mean (red bars and lines) at the experimental site at Changwu Agricultural Research Station, Shaanxi Province, China**.

Rainfall and temperature during the season are shown in **Figure 1**. In 2015, precipitation (197 mm) during the fallow period (July–September) was 31% less than the long-term mean (287 mm). Moreover, the growing season was warmer and drier than average. Temperatures from November 2015 to April 2016 were higher than the long-term mean, especially in March and April, which were 7.0 and 13.1°C, respectively, while the respective long-term averages were 4.3 and 10.7°C. Rainfall was lower than the long-term mean (304 mm), with 216 mm during the growing season (**Figure 1**), 29% less than the long-term mean. Precipitation from March to April (44 mm) was 39% less than the long-term mean (73 mm) and from May to June (89 mm) was 16% less than the long-term mean (106 mm), indicating that the experiment (if without irrigation) was subjected to moderate drought conditions before anthesis and after anthesis, respectively.

### Grain Yield and Yield Components

Under water stressed condition, CH1 and CH58 did not differ in grain yield and grain number, respectively, in the both experiments (**Table 1**). CH1 had significantly higher TKW than CW134 and CH58 (*P* < 0.05). CH1 also had the highest HI in both experiments (**Table 1**). In the rhizobox experiment, cultivar CH1 produced the highest grain yield when well-watered (*P* < 0.05). The higher spike number and grains number per unit area also found in CH1, and especially the TKW, which was the highest under well-watered treatment. The field experiment produced similar results (**Table 1**). Compared with the water-deficit treatments, yields of CW134, CH58 and CH1 in well-watered treatments increased by 58, 54, and 70%, respectively, in the rhizobox experiment; and by 14, 6.3, and 23%, respectively, in the field experiment (**Table 1**). These findings imply that grain yield in the newly released cultivar (CH1) has higher yield potential, but the modern cultivar (CH58) has greater yield stability.

**Table 1:** Grain yield and yield components of three wheat cultivars (CW134, CH58, and CH1) under moderate drought stress (MD) and well-watered conditions (WW; 2015 Rhizobox experiment), or under rainfed (RF) and irrigated (IR) conditions (2016 Field trial).

Treatments		Grain yield (g)	Spike number	No. of grains	TKW (g)	HI
**2015 Rhizobox experiment**						
MD	CW134	2.24 d	4.29 b	66.4 d	34.2 c	0.23 c
	CH58	2.39 c	4.80 b	73.6 c	33.7 c	0.23 c
	CH1	2.40 c	4.67 b	69.9 cd	36.1 b	0.29 a
WW	CW134	3.53 b	6.22 a	109 b	36.8 b	0.23 c
	CH58	3.69 b	5.33 ab	116 a	36.8 b	0.23 c
	CH1	4.09 a	5.89 a	119 a	38.2 a	0.26 b
Cultivar		^∗^	ns	^∗^	^∗^	^∗∗^
Water		^∗∗^	^∗∗^	^∗∗^	^∗^	ns
Cultivar × Water		^∗∗^	^∗∗^	^∗∗^	ns	^∗^

**2016 Field trial**						
RF	CW134	410 d	519 cd	896 d	45.8 d	0.28 c
	CH58	475 c	540 c	988 bc	46.4 d	0.31 b
	CH1	458 c	499 d	964 c	49.3 b	0.34 a
IR	CW134	483 c	531 c	1025 b	47.2 c	0.29 c
	CH58	511 b	565 b	1083 a	47.3 c	0.32 b
	CH1	567 a	629 a	1110 a	51.1 a	0.34 a
Cultivar		^∗^	^∗^	^∗∗^	^∗^	^∗^
Water		^∗∗^	^∗∗^	^∗^	^∗^	ns
Cultivar × Water		ns	ns	^∗∗^	ns	ns


*Data for rhizobox experiment were calculated for each rhizobox, and for the field trial on one square meter. TKW, thousand-kernel-weight; HI, harvest index. For each experiment, values in a column followed by the same letter are not significantly different at *P* < 0.05 as determined by the Duncan’s test. For ANOVA results in each experiment, ^∗^*P* < 0.05, ^∗∗^*P* < 0.01, ns, not significant.*

### Aboveground Growth

At the stem elongation stage, the higher tiller number per unit area and leaf area/leaf area index (LAI) of CH58 led to the highest shoot mass and lowest root-shoot ratio under both water treatments in both experiments (**Table 2**). At anthesis in the field experiment, water-stressed CH1 had the lowest shoot mass and tiller number while water-stressed CH58 had the lowest root-shoot ratio (**Table 3**). When well-watered, CH58 had the lowest shoot mass and tiller number per unit area. While CW134 and CH1 had similar tiller number per unit area, LAI, and shoot mass, as well as root-shoot ratio (**Table 3**). Well-watered plants of CW134, CH58 and CH1 had more shoot mass (9.5, 3.6, and 23%, respectively) than water-stressed plants (**Table 3**). This suggests that the shoots of the modern cultivar CH58 grew faster in the early season but were more stable than the other two cultivars by anthesis.

**Table 2:** Tiller number, leaf area, shoot mass, and root-shoot ratio of three wheat cultivars (CW134, CH58, and CH1) at the stem elongation stage under moderate drought stress (MD) and well-watered conditions (WW; 2015 Rhizobox experiment), or under rainfed (RF) and irrigated (IR) conditions (2016 Field trial).

Treatments		Tiller number	Leaf area (cm^2^)	Shoot mass (g)	Root-shoot ratio
**2015 Rhizobox experiment**					
MD	CW134	6.67 c	15.1 d	2.62 c	0.78 c
	CH58	5.33 d	16.6 c	3.26 bc	0.58 d
	CH1	5.67 d	15.7 cd	2.52 c	0.70 c
WW	CW134	8.33 b	20.4 b	3.87 b	1.36 a
	CH58	9.33 a	26.3 a	5.69 a	1.00 b
	CH1	7.33 c	27.4 a	3.99 b	1.24 a
Cultivar		^∗^	^∗∗^	^∗^	^∗^
Water		^∗^	^∗^	^∗∗^	^∗^
Cultivar × Water		ns	^∗∗^	^∗∗^	^∗∗^

**2016 Field trial**			**LAI (m^2^ m^-2^)**		
RF	CW134	1268 c	1.80 d	104 d	1.13 a
	CH58	1240 c	2.44 a	162 b	0.61 d
	CH1	1230 c	2.24 b	122 cd	0.81 b
IR	CW134	1353 b	2.09 c	146 b	0.83 b
	CH58	1418 a	2.35 ab	196 a	0.70 c
	CH1	1350 b	2.39 a	142 bc	0.78 bc
Cultivar		^∗^	^∗^	^∗^	^∗^
Water		^∗∗^	ns	^∗^	^∗∗^
Cultivar × Water		ns	^∗^	ns	^∗^


*Data for rhizobox experiment were calculated for each rhizobox, and for the field trial on one square meter. For each experiment, values in a column followed by the same letter are not significantly different at *P* < 0.05 as determined by the Duncan’s test. For ANOVA results in each experiment, ^∗^*P* < 0.05, ^∗∗^*P* < 0.01, ns, not significant.*

**Table 3:** Tiller number, leaf area index (LAI), shoot mass, and root-shoot ratio of three wheat cultivars (CW134, CH58, and CH1) at anthesis under rainfed (RF) and irrigated (IR) conditions in the field.

Treatments		Tiller number	LAI (m^2^ m^-2^)	Shoot mass (g)	Root-shoot ratio
**2016 Field trial**					
RF	CW134	964 c	1.47 b	1364 c	0.14 b
	CH58	939 cd	1.79 a	1354 c	0.11 c
	CH1	894 d	1.60 ab	1172 d	0.16 a
IR	CW134	1078 ab	1.92 a	1493 a	0.17 a
	CH58	1031 b	1.73 a	1403 bc	0.12 c
	CH1	1136 a	2.00 a	1446 ab	0.15 ab
Cultivar		^∗^	ns	^∗^	^∗^
Water		^∗^	^∗^	^∗^	^∗∗^
Cultivar × Water		^∗∗^	^∗^	^∗^	^∗^


*Data for field trial based on one square meter. Values in a column followed by the same letter are not significantly different at *P* < 0.05 as determined by the Duncan’s test. For ANOVA results, ^∗^*P* < 0.05, ^∗∗^*P* < 0.01, ns, not significant.*

### Root Mass and Root Length Density

**FIGURE 2:**
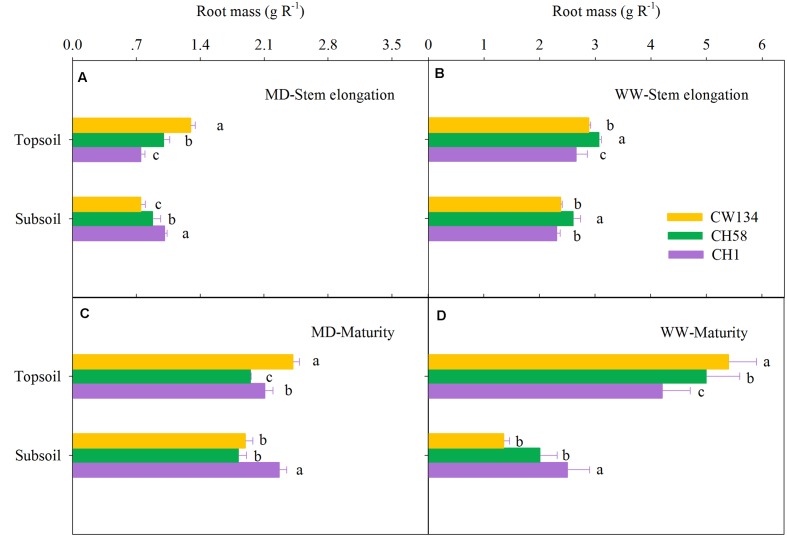
**Root mass in the topsoil and subsoil layers for CW134, CH58 and CH1 in the rhizobox experiment at** **(A,B)** stem elongation and **(C,D)** maturity under **(A,C)** moderate drought stress (MD) and **(B,D)** under well-watered (WW) conditions. Different letters in the same growth period imply a significant difference at *P* < 0.05. Horizontal bars represent + one standard error of the mean (*n* = 3).

**FIGURE 3:**
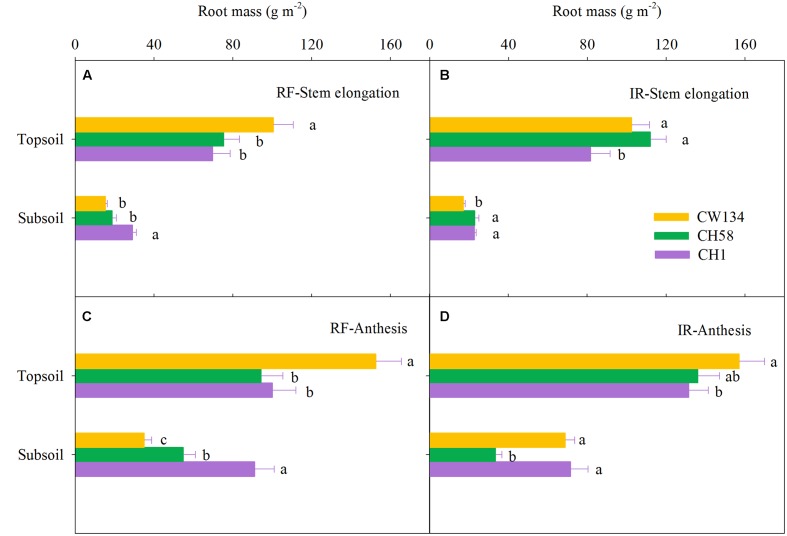
**Root mass in the topsoil and subsoil layers for CW134, CH58 and CH1 in the field trial at** **(A,B)** stem elongation and **(C,D)** anthesis under **(A,C)** rainfed (RF) and **(B,D)** irrigated (IR) conditions. Different letters in the same growth period imply a significant difference at *P* < 0.05. Horizontal bars represent + one standard error of the mean (*n* = 3).

**FIGURE 4:**
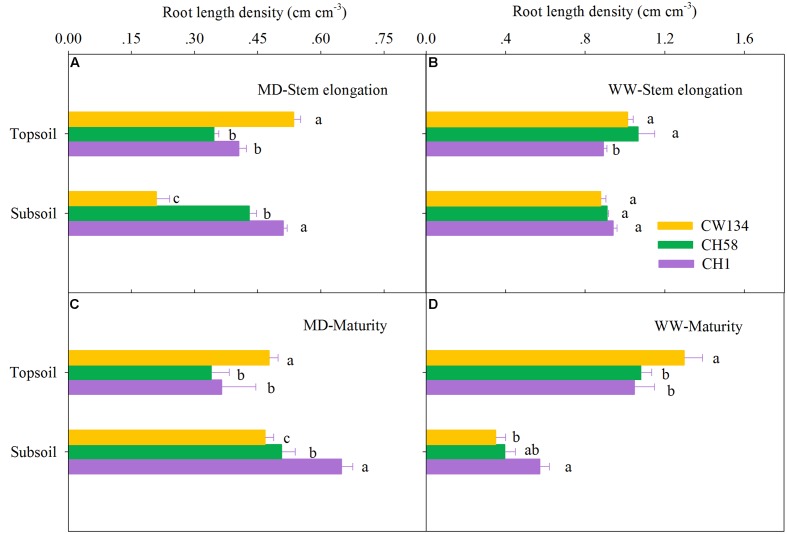
**Root length density in the topsoil and subsoil layers for CW134, CH58 and CH1 in the rhizobox experiment at** **(A,B)** stem elongation and **(C,D)** maturity under **(A,C)** moderate drought stress (MD) and **(B,D)** well-watered (WW) conditions. Different letters in the same growth period imply a significant difference at *P* < 0.05. Horizontal bars represent + one standard error of the mean (*n* = 3).

**FIGURE 5:**
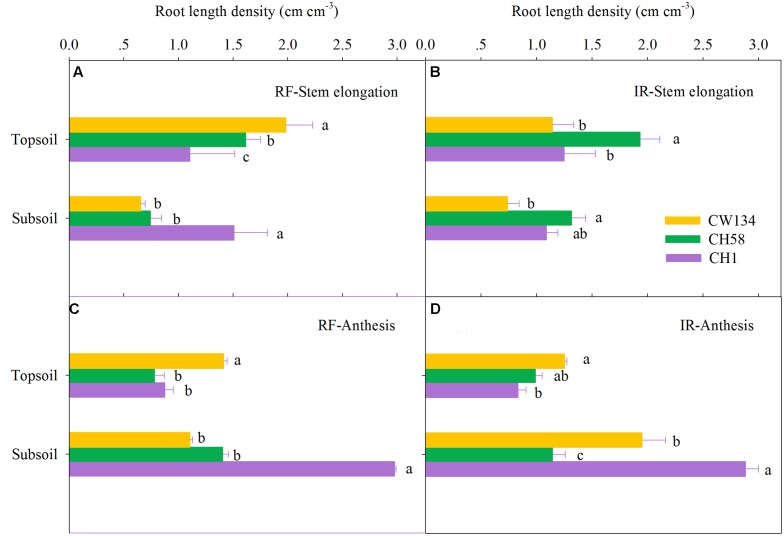
**Root length density in the topsoil and subsoil layers for CW134, CH58 and CH1 in the field trial at** **(A,B)** stem elongation and **(C,D)** anthesis under **(A,C)** rainfed (RF) and **(B,D)** irrigated (IR) conditions. Different letters in the same growth period imply a significant difference at *P* < 0.05. Horizontal bars represent + one standard error of the mean (*n* = 3).

Large variations in root mass (**Figures 2**, **3**) and root length density (**Figures 4**, **5**) were found between water treatments and among the three cultivars in the rhizobox experiment (**Figures 2**, **4**) and in the field trial (**Figures 3**, **5**). Under water stress, CW134 had the greatest topsoil (upper 20 cm) root mass and RLD during stem elongation, anthesis and maturity in the rhizobox (**Figures 2A,C**, **4A,C**) and field (**Figures 3A,C**, **5A,C**) experiments. CH1 had the highest subsoil root mass (below 20 cm in rhizobox experiment, 20–100 cm in field experiment) and RLD in the rhizobox (**Figures 2A,C**, **4A,C**) and field (**Figures 3A,C**, **5A,C**) experiments. In the field experiment, rainfed CH58 and CH1 had similar topsoil root mass and RLD at stem elongation and anthesis (**Figures 3A,C**, **5C**); At anthesis, rainfed CH58 had significantly higher subsoil root mass than CW134, but it was significantly lower than CH1 (*P* < 0.05) (**Figure 3C**); CW134 and CH58 had similar RLD at both stem elongation and anthesis (**Figures 5A,C**).

In both experiments, at stem elongation, well-watered CH58 had the highest root mass and RLD in the topsoil and subsoil (**Figures 2**–**5B**), and well-watered CH1 remained the lowest root mass and RLD in the topsoil, except RLD in field experiment (**Figures 2**–**5B**). However, at anthesis, CH58 and CW134 had similar topsoil root mass and RLD under well-watered treatments (**Figures 3D**, **5D**), and CH58 had the lowest subsoil root mass and RLD, which was significantly lower than the other two cultivars (**Figures 3D**, **5D**). CW134 and CH1 were similar in root mass (**Figure 3D**), but CH1 had significantly higher RLD than CW134 under irrigated condition (**Figure 5D**). These results indicate that, under water-deficit conditions, CW134 has the most topsoil root (root mass and RLD) and CH1 has the most subsoil root for the entire growth stage. Moreover, CH58 has smaller roots under water stress, but they grew better than other two cultivars in both soil layers when well-watered during early growth.

### Correlations between Root and Yield Traits

Simple correlation coefficients between root traits and grain yield and yield traits showed that significant negative correlations between topsoil root (mass and RLD) with grain yield in the two water treatments in rhizobox experiment (*P* < 0.01, **Table 4**). In contrast to the topsoil, subsoil root mass and RLD had significant positive correlations with grain yield in the water-stress treatments of both experiments (*P* < 0.05). Significant negative correlations between topsoil RLD and grain number were observed in well-watered and irrigated treatments in the field experiment (*P* < 0.05). When water limited, TKW and subsoil root mass were positively correlated in both experiments (*P* < 0.01, **Table 4**).

**Table 4:** Correlation coefficients between root and yield traits in three wheat cultivars (CW134, CH58, and CH1) under moderate drought stress (MD) and well-watered conditions (WW; 2015 Rhizobox experiment), or under rainfed (RF) and irrigated (IR) conditions (2016 Field trial).

Treatments			Grain yield	Spike number	No. of grains	TKW	HI
**2015 Rhizobox experiment**												
Root mass	Topsoil root	MD	-0.82**	0.44	0.16	0.01	0.05
		WW	-0.97**	0.90**	-0.24	-0.11	0.56
	Subsoil root	MD	0.92**	0.23	0.77*	0.83**	0.91**
		WW	0.25	-0.96**	0.48	0.16	-0.42
RLD	Topsoil root	MD	-0.56	0.39	0.72*	0.36	0.54
		WW	-0.75*	0.91**	-0.70*	-0.31	0.48
	Subsoil root	MD	0.82**	-0.25	0.35	0.44	0.49
		WW	0.95**	-0.84**	0.10	0.02	-0.78*

**2016 Field trial**												
Root mass	Topsoil root	RF	-0.87**	-0.02	-0.51	-0.76*	0.15
		IR	-0.60	-0.64	-0.47	-0.51	-0.52
	Subsoil root	RF	0.78*	-0.42	0.39	0.92**	0.03
		IR	0.24	0.21	-0.09	0.52	-0.08
RLD	Topsoil root	RF	-0.85**	-0.14	-0.76*	-0.48	0.17
		IR	-0.84**	-0.88**	-0.72*	-0.63	-0.72*
	Subsoil root	RF	0.75*	-0.64	0.34	0.90**	0.12
		IR	-0.30	-0.38	-0.42	-0.04	-0.47


*In the rhizobox experiment, root samples were collected in 2015 at maturity while in the field experiment, they were collected in 2016 at anthesis. RLD, root length density. For ANOVA results in each experiment, ^∗^, ^∗∗^, Significant at *P* < 0.05 and 0.01, respectively.*

The above findings imply that root traits significantly influenced grain yield and its components. With water deficit, more roots in the topsoil layer and lower RLD in the subsoil layer resulted in lower grain yields. With sufficient water, higher RLD in the topsoil resulted in fewer grains per unit, while more subsoil root mass under water stress resulted in higher TKW, which may increase grain yield.

### Soil Water Content and Water Consumption

**FIGURE 6:**
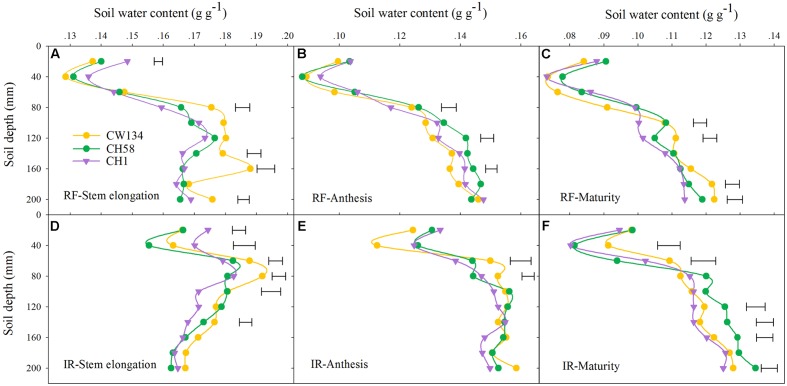
**Soil water content of CW134, CH58 and CH1 in the field trial at** **(A,D)** stem elongation, **(B,E)** anthesis and **(C,F)** maturity under **(A–C)** rainfed (RF) and **(D–F)** irrigated (IR) conditions. Horizontal bars represent l.s.d at *P* < 0.05 (*n* = 3).

In the field experiment, CH1 had significantly higher soil water content in the topsoil layer (0–20 cm) at stem elongation than CW134 and CH58 in both treatments, but the three cultivars did not differ at later growth stages (**Figure 6**). In subsoil layers (20–100 cm), CW134 had a high soil water content at stem elongation, especially below 80 cm (rainfed) and 60 cm (irrigated) (**Figures 6A,D**). At anthesis, the soil water content at below 100 cm profile was the least in CW134 and the highest in CH58 under water stress (**Figure 6B**), but no significant difference of soil water content was found among three cultivars under irrigated condition (**Figure 6E**). At maturity, CH1 had the lowest soil water content at 100–200 cm and CW134 was the highest under rainfed (**Figure 6C**). When under irrigation, CH58 had the highest soil water content in the soil profile below 100 cm (**Figure 6F**), with more deep soil water left at the end of the season.

**FIGURE 7:**
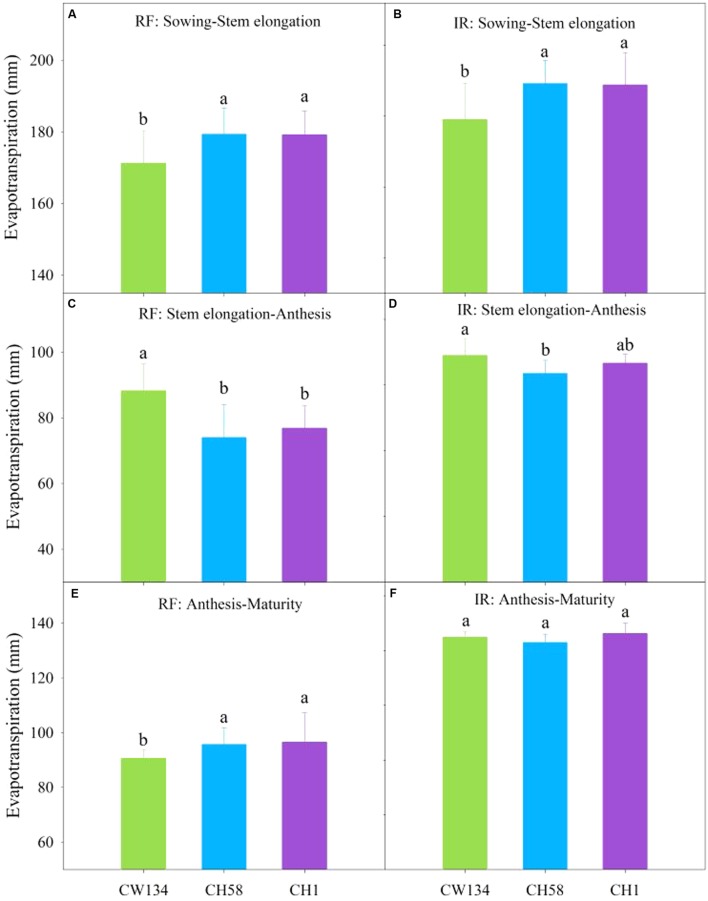
**Evapotranspiration (ET) of CW134, CH58 and CH1 in the field trial from** **(A,B)** sowing to stem elongation, **(C,D)** stem elongation to anthesis and **(E,F)** anthesis to maturity under **(A,C,E)** rainfed (RF) and **(B,D,F)** irrigated (IR) conditions. For the same growth period, bars with different letters are significantly different (*P* < 0.05). Data are means + standard error (*n* = 3).

The old cultivar CW134 had lower ET than the other two cultivars just before stem elongation in both rainfed and irrigation treatments (*P* < 0.01) (**Figures 7A,B**), but the reverse was true from stem elongation to anthesis (**Figures 7C,D**). After anthesis, the newly released cultivar CH1 used more water than other two cultivars under rainfed treatment (**Figure 7E**), but ET did not differ among three cultivars when under irrigated treatment (**Figure 7F**). Over the entire growing cycle, water use did not differ among the three cultivars in the rainfed treatment (350, 349, and 352 mm in CW134, CH58 and CH1, respectively); however, when well-watered, CH58 consumed less water (79 mm) than CH1 (85 mm) and CW134 (82 mm).

These findings clearly demonstrate that the cultivars changed their water consumption throughout the growing period. The old landrace cultivar CW134 used less soil water before stem elongation than the other two cultivars, but used more water between stem elongation and anthesis stages, leading to a lower soil water content after anthesis. However, CH58 and CH1 used less water during vegetative growth and more after anthesis, especially from subsoil layers.

## Discussion

### Grain Yield, Yield Potential, and Yield Stability

This study found that the newly released cultivar CH1 and the modern cultivar CH58 produced significantly higher grain yield than that of the old landrace CW134. The higher grain yield in CH1 was primarily attributed to a greater TKW and in CH58 to more grains per unit area, which is the yield component responsible for increases in wheat grain yield (Siddique et al., 1989a; Hall and Richards, 2013). The results presented here show that the newly released cultivar CH1 has a higher yield potential, and increases in yield potential may correlate with the higher HI (Foulkes et al., 2007; del Pozo et al., 2016). Selection for higher yields in non-stressed environments has indirectly increased grain yield in many drought stress environments (Cattivelli et al., 2008). Nevertheless, further breeding is required to improve traits for better yield potential in water-stressed conditions. Positive correlations have been found between grain yield and yield potential under drought stress and non-stressed environments (Tester and Langridge, 2010; Mohammadi et al., 2011). However, indirect selection of mean yield and yield potential genotypes under non-stressed environments may not be an appropriate choice for water-stressed environments (Abdolshahi et al., 2013). Compared with CW134 and CH1, the grain yield of the modern cultivar CH58 was more stable. Yield improvement has been associated with increased stress tolerance, which is thought to result from selection for yield stability (Tollenaar and Lee, 2002). In semi-arid area, water limitation is a crucial factor affecting crop yield. Simane et al. (1993) suggested that yield stability is a better indicator of drought resistance than yield potential. Genotypes with high yield stability should be planted in the regions with low-input systems and high yield potential in regions with high-input systems (Calderini and Slafer, 1999). The grain yield of rainfed system achieving some 5000 kg hm^-2^ may be considered as a high input system (Song et al., 2009). In the field, winter wheat grown in the year with less rainfall than the average during the growing season could suffer drought stress causing the reduced grain yield. However, irrigation with 90 mm of water significantly increased the yield of three cultivars. Hence, the newly released cultivar (CH1), with higher yield potential, should be more suitable for the semi-arid Loess Plateau, while the modern cultivar (CH58) with higher yield stability may be planted in a dry area with lower input (no irrigation).

### Root and Aboveground Growth Alteration as Adaptation to Drought Stress

A better understanding of root systems is critical to crop improvement in water-limited environments. A vigorous root system in early growth stage, with significant root mass and RLD (Palta et al., 2011), has advantages early in the growing season due to its ability to capture more water and facilitate crop establishment and growth such as faster leaf area development and shoot biomass increment (Rebetzke and Richards, 1999; Liao et al., 2006). The modern cultivar CH58, which had higher root mass and RLD during stem elongation, could be considered as an ‘early vigor’ genotype under well-watered condition. The lower ET of CH58 from stem elongation and anthesis may due to the greater leaf area and shoot mass, which has thought to shade the soil surface and reduce water loss through soil evaporation (Botwright et al., 2002).

In the present study, root mass and RLD in the topsoil layer had a significant negative correlation with grain yield. Greater root mass, especially in topsoil layer, increases inter-root competition and delays the effectiveness of roots in capturing resources under water-stress conditions (Ma et al., 2008; Fang et al., 2011). As a consequence, an overabundance topsoil root mass may acquire more soil water for plant growth (King et al., 2003). A study in Australia found that, compared with old cultivars, modern cultivars had more grain yield and less root mass in top 40 cm layers (Siddique et al., 1990; Aziz et al., 2016). In our study, the old landrace cultivar CW134 had the most roots (root mass and RLD) in the topsoil layer and used more water from stem elongation to anthesis, making less soil water available to plants after anthesis, which inevitably affected grain filling. Meanwhile, the paucity of roots in the subsoil layer could not fully explore soil water in deep layer, which may explain why CW134 had the lowest grain yield. Passioura (1983) reported that less root mass in the topsoil layer would be beneficial only if more water could be utilized in deep soil layers.

The newly released cultivar CH1 at anthesis under drought stress had the lowest shoot mass, tiller number and leaf area per unit area. Tiller number is considered as a function of competition for light and nitrogen (Paynter and Hills, 2009). In field experiment, rainfall (44.4 mm) was 39% less than the long-term mean (72.8 mm) during vegetative growth, indicating that the plants were subjected to moderate drought conditions. CH1 with fewer tillers reduced soil water uptake between stem elongation and anthesis, and possibly reduced intra-plant competition simultaneously. Our results showed that CH1 had less root mass in the topsoil layer and less total root mass, but more root mass and higher RLD in the subsoil layers in both the nursery and field experiments. This resulted in greater extraction of water by maturity, especially at depths below 100 cm in the field. A higher root mass and RLD are critical for increased early vigor and pre-anthesis water use, which would improve grain yield in wheat crops which rely mainly on seasonal rainfall (Rebetzke and Richards, 1999; Liao et al., 2004). In our findings, large subsoil layer roots have positive effects on yield and yield components, especially under water stress. Higher root distribution at depth and higher RLD in the subsoil layers are considered potential traits for the adaptation of wheat to water stress, increasing the water extraction capacity in the subsoil profile for grain filling and increased grain yield (King et al., 2003; Palta et al., 2011), especially under terminal drought stress (Passioura, 1983; Gaur et al., 2008). Angus and van Herwaarden (2001) argued that, if subsoil water can be full exploitation between anthesis and grain filling, the grain yield will be increased significantly under drought stress. Lilley and Kirkegaard (2007) suggested that deep soil water is more valuable in above-average rainfall seasons because of a more efficiently conversion of deep soil water to grain. Compared with old landrace CW134, modern cultivar CH58 and newly released cultivar CH1 consumed more soil water during anthesis and maturity under drought stress condition. Each additional millimeter of water during grain filling produced 55 kg hm^-2^ of grain at harvest (Manschadi et al., 2006). That’s why higher yield were observed in both CH58 and CH1 when suffered drought stress. For CH1, more roots in subsoil and less water left in deep layer at maturity implied that the soil water could be fully exploitation, especially when soil water improved. Kirkegaard et al. (2007) found that each additional millimeter of water extracted from the subsoil (e.g., 1.35–1.85 m) after anthesis improved 62 kg hm^-2^ yield under post-anthesis water stress This may explain that why a higher yield potential had found in the newly released cultivar CH1. Indeed, CH1 had the highest TKW which was positively correlated with subsoil root mass and RLD. CH1 reduced water use during vegetative growth so that more soil water was available after anthesis, particularly at depth. Our research is consistent with those of Arai-Sanoh et al. (2014), who found that cultivars with deep rooting produced more grain yield, which was mainly due to increased TKW and resulted in a higher HI. In addition, our findings indicate that CH58, with the lowest root mass and RLD at anthesis under irrigation, did not maximize the extraction of water in the soil profile, and more water was left unused at the end of the season. This may explain the lower yield in CH58 when soil moisture improved, and that grain yield was more stable under different water conditions.

## Conclusion

The present study showed that the three wheat cultivars have different root properties under well-watered and water stressed environments. Root system adaptive to drought stress was improved along with genetic improvements. The old cultivar (CW134) produced more topsoil root mass and less subsoil root mass throughout the growing season, which restricted access to water in the subsoil and thus limited grain yield. In contrast, the newly released cultivar (CH1) simulated greater amount of root growth in subsoil enabling access to water, especially when the topsoil was drying after anthesis, leading to a higher yield potential.

## Author Contributions

YF, YD, and YC designed the experiments and performed data analyses. YF and JW performed the nursery experiment. YF, AW, and SQ performed the field experiment. YF, YD, BX, SZ, KS, and YC contributed to the writing and revision. All authors have read and approved the final manuscript.

## Conflict of Interest Statement

The authors declare that the research was conducted in the absence of any commercial or financial relationships that could be construed as a potential conflict of interest.
